# Overlooking the Obvious during the COVID-19 Pandemic: Dyspnoea with Asymmetric Breath Sounds in a Toddler

**DOI:** 10.1155/2021/8855962

**Published:** 2021-02-02

**Authors:** Heli Salmi, Heini Harve-Rytsälä, Paula Rautiainen, Sari Pyörälä, Johanna Hästbacka

**Affiliations:** ^1^Paediatric Research Center, New Children's Hospital, University of Helsinki and Helsinki University Hospital, Helsinki, Finland; ^2^Department of Anaesthesia and Intensive Care, New Children's Hospital, University of Helsinki and Helsinki University Hospital, P.O. Box 347, Fi-00029 HUS, Finland; ^3^Emergency Medicine and Services, University of Helsinki and Helsinki University Hospital, P.O. Box 340, FI-00029 HUS, Finland; ^4^Department of Paediatric Surgery, New Children's Hospital, University of Helsinki and Helsinki University Hospital, P.O. Box 347, Fi-00029 HUS, Finland

## Abstract

**Background:**

Paediatric healthcare specialists are concerned about the secondary effects of the COVID-19 pandemic on children. We report a case of acute respiratory distress in a healthy toddler whose healthcare providers were sidetracked from the correct diagnosis by suspicion of COVID-19. *Case Presentation*. The patient was a 20-month-old healthy boy. In the morning, he had coughed while drinking milk. He was asymptomatic for the day but presented with acute respiratory distress when lying down in the evening. An ambulance was called, and he was taken to a tertiary hospital's paediatric emergency department, where his condition and oxygen saturation fluctuated. He had mildly elevated temperature and petechiae on his trunk, showed asymmetrical radiographic and auscultatory pulmonary findings, and did not tolerate any exertion. Pneumonia was suspected, SARS-CoV-2 was considered as potential causative agent, and the child was admitted to a Paediatric Intensive Care Unit. As the patient did not show clear signs of infection or bronchial obstruction, the events were thoroughly rediscussed with the caregiver next morning. It was then found out that the child had also been eating cashew nuts. Multiple pieces of cashew nuts were removed from the left bronchial tree in a bronchoscopy. After the procedure, all symptoms promptly resolved. Foreign body aspiration—an obvious cause of acute respiratory distress in our patient's age group—was overlooked by experienced emergency medical care providers and paediatric critical care physicians due to the slightly unusual presentation, incomplete anamnestic information, and a bias to consider COVID-19 in the current exceptional circumstances.

**Conclusions:**

Emergency care providers are instructed to consider all patients with respiratory distress as potential COVID-19 patients. However, the clinical course of COVID-19 infection is usually mild in children. Therefore, alternative causes for serious breathing difficulty are more likely, and all differential diagnoses should be considered in the usual unbiased manner.

## 1. Introduction

Children seem to be spared from the most serious forms of coronavirus disease 2019 (COVID-19) [1–4] caused by the novel pandemic coronavirus SARS-CoV-2. However, cases with significant symptoms and need for hospitalization including intensive care have been reported [2, 3]. Coinfection with other airway pathogens, such as *Mycoplasma pneumoniae* and respiratory syncytial virus, has been reported in 6.5% [5] or as much as 51% [6] of children with COVID-19. Furthermore, since other infectious and noninfectious causes for dyspnoea and respiratory failure are more common in children than SARS-CoV-2, it is crucial to carefully evaluate all differential diagnoses in children with respiratory symptoms. The continuous presence of COVID-19 in media may create an illusion of high prevalence of COVID-19 among children. Thus, diagnosis and treatment of common conditions may be delayed if caregivers avoid medical service contacts for fear of contracting COVID-19 and if healthcare professionals prioritize COVID-19 at the cost of delaying the diagnosis and treatment of more common conditions.

We report a case of rapidly deteriorating hypoxemic respiratory compromise in a 20-month-old toddler during the COVID-19 pandemic. We recognize that his prehospital and in-hospital treatments were probably influenced by the pandemic causing diagnostic delay.

## 2. Case Presentation

The patient was a previously healthy 20-month-old boy. He had mild atopic eczema, with no history of asthma or wheezing in respiratory infections. He had been vaccinated according to the Finnish vaccination schedule, which includes vaccinations against *Haemophilus influenzae*, *Bordetella pertussis*, *Streptococcus pneumoniae*, and influenza. He had started day care the previous week. His parents and elder sibling were well and asymptomatic.

On the morning of the hospitalization, the boy had suddenly started to cough vigorously while drinking milk at breakfast. The coughing had ceased, and the boy had had an uneventful day at day care, playing, eating, and napping normally. In the evening, he had been irritable and reluctant to eat. He had gone to bed as usual but soon woken up with cough and apparent dyspnoea. His father had called an ambulance when noticing that he had turned blue in the face.

At arrival of the ambulance, the child was dyspnoeic, the oxygen saturation was 90%, and the axial temperature was 38.5 C. The boy met the emergency medical services (EMS) criteria for suspicion of COVID-19, and he was transported to the emergency room (ER) with a notion “suspicion of COVID-19” and with the EMS personnel wearing personal protective equipment (PPE) according to the protocol.

**Figure 1:**
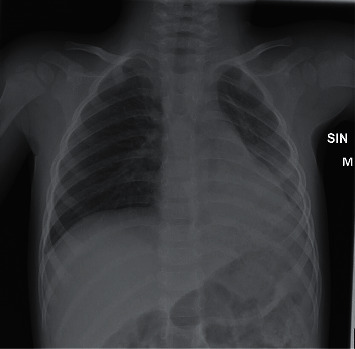
Chest X-ray at arrival, showing infiltrates and/or atelectasis in the left lower lobe.

Upon arrival at the ER, nasal high flow (NHF) oxygen was started and the patient was provided salbutamol by inhalation. A nasopharyngeal swab was taken for a SARS-CoV-2 RNA test. The patient's oxygen saturation had risen to over 95% but dropped to 56% while establishing an intravenous line, as he became agitated. A paediatric critical care specialist was called in, and preparations for emergency intubation were started. The team dressed in full PPE. With 80–100% oxygen to NHF with 25 l/min flow (2 l/kg), the child was still breathing heavily with prolonged expiration but had an oxygen saturation of 93%, with occasional desaturations to 60–70% while agitated. Breath sounds were absent from the left lower lung field. There were no rales or wheezing. The child was tachycardic (124–157/min) and febrile (38.3 C), his capillary refill time was just in the normal range (3 s), peripheral pulses were well palpable, and blood pressure was normal (110/79 mmHg). The child was conscious but lethargic, and petechiae were noted on his chest. A chest X-ray showed a diffuse opacity in the left lower lobe (Figure 1). The heart was not enlarged, and there was no pulmonary congestion. The laboratory findings, showing an elevated white cell count and hypoxaemia, are summarized in Table 1.

**Figure 2:**
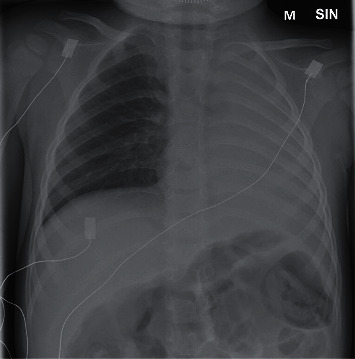
Follow-up chest X-ray 12 h after arrival. The left lung is completely atelectatic, and the mediastinum has shifted to the left.

An intravenous fluid bolus was given, blood cultures were obtained, and intravenous ceftriaxone was administered. The child was transferred to a negatively pressurized isolation room in the Paediatric Intensive Care Unit (PICU). His breathing remained adequate on NHF with 70% FiO_2_ and dexmedetomidine sedation. Arterial blood gases and lactate were in the normal range (Table 1). A bedside ultrasound examination showed a consolidated lung on the left side with no pleural effusion and normal heart contractility. During the ensuing 8 hours, there were no new organ dysfunctions, suggestive of sepsis, nor coughing or mucus secretions suggestive of pneumonia. However, in a follow-up chest X-ray, the whole left lung was atelectatic and the heart had shifted left (Figure 2). Breath sounds were absent on the left side.

The following morning, a reassessment of the rapidly evolved hypoxemic respiratory insufficiency with single lung involvement and without symptoms of an infection apart from mild fever led to a second interview with the mother. This revealed that in addition to drinking milk, the patient had also been eating cashew nuts.

**Figure 3:**
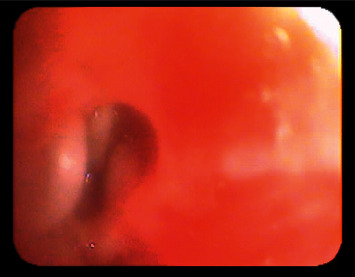
A still frame from the bronchoscopy, showing the foreign material in the left main bronchus.

**Figure 4:**
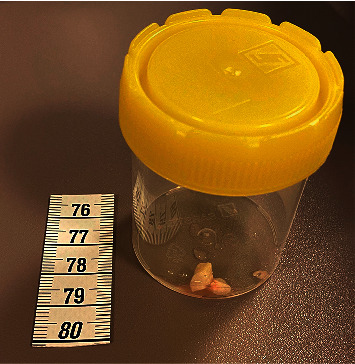
Two pieces of cashew nuts extracted from the left main bronchus.

A bronchoscopy was scheduled, and two large and several smaller pieces of cashew nuts were removed from the left main bronchus and the left upper lobe bronchus (Figures 3 and 4) two hours later. The atelectasis vanished, supplementary oxygen and respiratory support were promptly weaned, and the child was extubated on the same day. The SARS-CoV2 test was negative. There were no new petechiae; the ones noticed the previous evening were probably due to forceful coughing, or perhaps due to scratching his atopic eczema, as the mother suggested. The boy made a full and brisk recovery.

## 3. Discussion and Conclusions

Although COVID-19 appears to be generally mild or asymptomatic in children [1–3], the medical care children receive is negatively influenced by COVID-19 control measures [7–9]. As patient flows and customary protocols are abruptly modified and massive media coverage results in a generalized attention shift, healthcare professionals may make diagnostic deviations by considering COVID-19 over other more probable diagnoses. Furthermore, parents may not dare to bring even severely ill children to the hospital for fear of contracting COVID-19. Consequently, paediatric healthcare providers have repeatedly expressed their concern about children becoming “secondary victims” of the COVID-19 pandemic [7–9].

Bronchial foreign body aspiration is a common cause of acute respiratory distress in children, particularly those aged three years or under. The left bronchus is affected less often than the right one. Diagnosis is often delayed due to the frequent symptomless period following the aspiration. The obstruction of the airway may worsen due to gradual swelling of the mucosa or swelling of the obstructing foreign object [10–12].

During a local epidemic peak of COVID-19 in the Helsinki University Hospital region, the prevalence of COVID-19 in children requiring EMS or ER care for infectious symptoms has been very low. Between 20/4/2020 and 20/5/2020, EMS contacted 358 children (0–15 years), of which 31 (3.7%) presented with symptoms leading to a SARS-CoV-2 RNA test. All 31 tests turned out negative. In a separate analysis for prevalence of SARS-CoV-2 in children requiring secondary or tertiary ER, 3/113 (2.7%) children (0–15 years) contacting a HUS ER with infectious symptoms between 8/4/2020 and 15/4/2020 were positive for SARS-CoV-2 RNA. These cohorts are small but regionally inclusive as HUS is the only provider of EMS as well as secondary- and tertiary-level ER care for children in the area. Consequently, even during a local epidemic peak of COVID-19, diagnostic measures should not overlook other common aetiologies for acute respiratory distress in order to avoid unnecessary delays in treatment.

In this case, EMS, ER, and PICU personnel—all dressed up in full PPE against COVID-19—consecutively overlooked a probable diagnosis in any toddler with acute onset dyspnoea, unilateral absence of breathing sounds, and unilateral lung atelectasis. The COVID-19 preparedness including PPE and alterations in usual protocols certainly caused an attention shift in the experienced professionals even when COVID-19 was not necessarily suspected as the primary diagnosis. The diagnosis may also have been delayed as the aspiration had not occurred to the conventional right side and as the patient displayed petechiae.

To conclude, it is valuable to note that foreign objects are not always aspirated to the right bronchus; petechiae can exist without sepsis; and in case of a critically ill child, any exceptional circumstances, such as those during the COVID-19 pandemic, should not distract from paying attention to the typical and critical aetiologies.

## Figures and Tables

**Table 1:** Laboratory values at arrival at night and the next morning.

Laboratory test	Source	Result at arrival	Result 6 h after arrival
Haemoglobin (g/L)	Blood	131	121
White cell count (10^9^/L)	Blood	21.5 (H)	13.9
Neutrophil count (10^9^/L)	Blood	16.10	10.56
Platelet count (10^9^/L)	Blood	344	266
C-reactive protein (mg/dL)	Plasma	<5	13 (H)
Glucose (mmol/L)	Blood	8.6	6.6
Lactate (mmol/L)	Arterial blood	0.7	0.6
pH	Arterial blood	7.40	7.39
Bicarbonate, standardized (mmol/L)	Arterial blood	23	23
Base excess (mmol/L)	Arterial blood	−2.3	−1.5
Oxygen, partial pressure (kPa)	Arterial blood	10.0 (L)	15.9 (H)
Carbon dioxide, partial pressure (kPa)	Arterial blood	4.8	5.1
Prothrombine time (% of normal)	Arterial blood	—	74

H (high) indicates a value higher than normal and L (low) indicates lower.

## Data Availability

Full laboratory findings are available from the authors upon request.

## Abbreviations

COVID-19: Coronavirus disease 2019
ED: Emergency department
EMS: Emergency medical services
ER: Emergency room
HUS: Helsinki University Hospital
PICU: Paediatric Intensive Care Unit
PPE: Personal protective equipment
SARS-CoV-2: Severe acute respiratory syndrome coronavirus 2.
